# Knowledge, attitudes and practices about human African trypanosomiasis and their implications in designing intervention strategies for Yei county, South Sudan

**DOI:** 10.1371/journal.pntd.0006826

**Published:** 2018-10-01

**Authors:** Salome A. Bukachi, Angeline A. Mumbo, Ayak C. D. Alak, Wilson Sebit, John Rumunu, Sylvain Biéler, Joseph M. Ndung'u

**Affiliations:** 1 Institute of Anthropology, Gender and African Studies, University of Nairobi, Nairobi, Kenya; 2 Research and Development, Passion Africa Limited, Nairobi, Kenya; 3 South Sudan Coordination Office, Malteser International, Juba, Republic of South Sudan; 4 Preventive Health Services, Ministry of Health, Juba, Republic of South Sudan; 5 Neglected Tropical Diseases, Foundation for Innovative New Diagnostics, Geneva, Switzerland; Institute of Tropical Medicine, BELGIUM

## Abstract

**Background:**

A clear understanding of the knowledge, attitudes and practices (KAP) of a particular community is necessary in order to improve control of human African trypanosomiasis (HAT).New screening and diagnostic tools and strategies were introduced into South Sudan, as part of integrated delivery of primary healthcare. Knowledge and awareness on HAT, its new/improved screening and diagnostic tools, the places and processes of getting a confirmatory diagnosis and treatment are crucial to the success of this strategy.

**Methodology:**

A KAP survey was carried out in Yei County, South Sudan, to identify gaps in community KAP and determine the preferred channels and sources of information on the disease. The cross-sectional KAP survey utilized questionnaires, complemented with key informant interviews and a focus group discussion to elicit communal as well as individual KAP on HAT.

**Findings:**

Most (90%) of the respondents had general knowledge on HAT. Lower levels of education, gender and geographic locations without a history of HAT interventions were associated with incorrect knowledge and/or negative perceptions about the treatability of HAT. Symptoms appearing in the late stage were best known. A majority (97.2%) would seek treatment for HAT only in a health centre. However, qualitative data indicates that existing myths circulating in the popular imagination could influence people’s practices. Seventy-one percent of the respondents said they would offer social support to patients with HAT but qualitative data highlights that stigma still exists. Misconceptions and stigma can negatively influence the health seeking behaviour of HAT cases. In relation to communication, the top preferred and effective source of communication was radio (24%).

**Conclusion:**

Gaps in relation to KAP on HAT still exist in the community. Perceptions on HAT, specifically myths and stigma, were key gaps that need to be bridged through effective education and communication strategies for HAT control alongside other interventions.

## Author summary

Misconceptions about sleeping sickness, a neglected tropical disease transmitted by tsetse flies, can be a hindrance to effective implementation of control interventions especially in the face of accelerating work to eliminate the disease. Understanding community knowledge, attitudes and practices about sleeping sickness is important in developing appropriate material for educating and sensitizing communities at risk of the disease. We conducted a study to establish community knowledge, attitudes and practices, including preferred channels of disseminating sleeping sickness information. Despite the fact that the community in Yei County knew about the disease, existing myths and stigma have the potential of influencing their health seeking behaviour.

The radio, community health workers and village elders were the most preferred sources of sharing information with the community. There is need to develop education and awareness material to address issues of existing myths, potential stigma, treat ability of HAT, importance of testing and treatment, as well as provide information on the new/improved testing and treatment approaches for HAT. In addition, this should be provided through use of preferred and trusted sources of information dissemination, which is critical in uptake of HAT control, management and prevention activities.

## Introduction

Human African trypanosomiasis (HAT), also known as sleeping sickness, is a parasitic neglected tropical disease of public health significance that mostly afflicts poor populations in endemic areas of rural Africa [1]. It is caused by two species of *Trypanosoma*: *T*. *brucei rhodesiense*, mostly found in eastern and southern Africa and causes the acute form of the disease, and *T*.*b*. *gambiense*, found in west and central Africa, and causes the chronic form of the disease. The disease occurs in two stages, early and late, and if not diagnosed early, affects the central nervous system causing severe neurological disorders, which can subsequently lead to death [2]. During the past decade, the number of HAT cases reported to the World Health Organization (WHO) per year has been falling progressively, arising from a concerted campaign to control the disease at international and country levels[3]. South Sudan is one of the countries that is still reporting cases of HAT [1]. According to [4],the disease is endemic in the southern and southwestern regions of South Sudan, near the borders with Uganda, the Democratic Republic of the Congo (DRC) and the Central African Republic (CAR). In the years 2011 and 2012, the number of cases reported in South Sudan was 272 and 317 respectively, which was the third highest behind the DRC and CAR [5]. Recurrent outbreaks of the disease in this country have been attributed to reduced control measures and/or socio-political crisis [6]. About 250,000 km^2^in South Sudan is infested with the tsetse fly vector, with about 1–2 million people at risk of HAT. *Glossina fuscipes fuscipes* is the sole vector for HAT in the three Equatoria States; Eastern Equatoria State, Central Equatoria State and Western Equatoria State. Yei county is one of the 10counties in Central Equatoria State and among the nine that are endemic for HAT[6].

Over the years, HAT activities in South Sudan were mainly carried out by NGOs but after 2006, when the numbers of new cases begun to decrease, most NGOs stopped or reduced their HAT related activities. This was due to difficulties in securing funding for what was no longer perceived as a serious problem [6, 7]. Currently a project in South Sudan has brought together the Government of South Sudan, Malteser International and Foundation for Innovative New Diagnostics (FIND), to intensify surveillance and control of HAT in the country in a sustainable manner, through the introduction and use of new screening and diagnostic tools and strategies as part of an integrated delivery of primary healthcare[8].The HAT rapid diagnostic test (RDT) is part of the new screening and diagnostic tools for HAT. If one tests HAT RDT positive, they are referred to the nearest facility for parasitological confirmation, including microscopy. If found positive by microscopy, the patient is staged and treated for HAT [9].Besides availability of new screening and diagnostic tools, well–trained staff and well-equipped health centers, accurate diagnosis and early identification of cases also depends on other community aspects, including knowledge, attitudes and practices (KAP).A study in north western Uganda recommended that the success of community-based interventions against tsetse depend on early engagements with communities and carefully designed sensitization campaigns that reach all communities [10].An understanding of community KAP therefore has relevance for disease control programmes and has been instrumental in revealing the level of knowledge, and misconceptions or misunderstandings, that could present obstacles to control intervention activities and potential barriers to behaviour change[11]. They help to suggest intervention strategies that reflect specific local circumstances and the cultural factors that influence them, and plan activities that are suited to the respective population involved [12].

Knowledge, attitudes and practices surveys are frequently used in health-seeking behaviour research with knowledge being assessed in order to see how far community knowledge corresponds to biomedical concepts[12]. A limitation in these kinds of studies is that other types of knowledge tend to be highly neglected with very little information being sought on knowledge about the health system [13]. Further, attitudes are never easy to obtain from such a survey while questions on practices are usually hypothetical, hence hardly permitting statements about actual practices or taking into consideration underlying contextual factors, hence affecting their reliability[14]. Besides the limitations of KAP surveys, they are very useful for assessing distribution of community knowledge in large-scale and for evaluating changes in knowledge after education and media campaigns. They permit rapid assessments, yielding quantitative data, and are therefore a cheap way to gain quick insights into main knowledge data[13].

Combining both qualitative and quantitative methods can address some of the limitations of each method (eg qualitative methods can help identify potential respondent bias present in KAP and inappropriate framing of questions, while quantitative surveys can collect data at scale). Both qualitative and quantitative studies play a role in highlighting information on community knowledge, perceptions and acceptance of HAT related control interventions. Qualitative studies on HAT in Uganda, South Sudan and the DRC have been useful in highlighting the importance of understanding community attitudes and perceptions in effective engagement in HAT interventions [10, 15,16].Similarly, a number of surveys on community’s KAP on HAT have been carried out in various endemic countries, including Tanzania [17], the DRC [18] and Nigeria[19]. These studies highlight people’s knowledge about cause, symptoms and prevention of HAT, people’s beliefs about the disease and where they would seek for treatment. They also highlight the notion that knowledge about tsetse and HAT can help communities understand and support tsetse and HAT control interventions. Given that there is a dearth of KAP studies in South Sudan, this survey sought to establish gaps in KAP of the community in Yei county in relation to HAT, and to identify the preferred channels and sources of information for HAT. The aim was to gather relevant information that would be used to develop an effective communication strategy and community awareness programme, through information education and communication (IEC) campaigns to run alongside the passive screening and diagnosis strategy recently introduced in the country.

## Methods

### Ethical statement

Ethical clearance for this study was granted by the Ministry of Health, Republic of South Sudan before commencement of activities. Prior to the interviews and discussions, all the eligible participants were consented individually. At that time the objectives, procedures, benefits and discomforts of the study were explained and they were assured of confidentiality. Their voluntary participation was recorded through a thumb print or signature before the interviews were conducted.

### Study area

Yei county is located southwest of Juba, South Sudan and lies close to the border of Uganda and the Democratic Republic of Congo (DRC). It has an area of 6,730 km^2^,is administratively divided into five payams, including Yei town, Otogo, Lasu, Mugwo and Tore. It is further divided into 22 Bomas and 100 villages, and has 31 health facilities and 74 educational facilities. The 2015–2020 projections indicate the human population of Yei county to be about 267,656, with 33,393 households [20]. The majority socio-linguistic groups are the Kakwas followed by Bari. Others include Avukaya, Pojulu, Kuku, Mundu and Keliko. The area receives adequate rainfall all year round and is suitable for food and cash crop farming. The main occupations of the inhabitants include farming, livestock farming and fishing.This social survey was carried out from 17^th^ November to 9^th^ December, 2015.

### Study design and data collection methods

The baseline KAP survey (hereafter referred to as survey)reported here involved a cross-sectional descriptive research design utilising mixed methods, whose unit of analysis was the individual participant. Each of the five payams (districts) in Yei county were included in the sample. Smaller units, that is, the boma/village in each of the payams, formed the units from which systematic sampling of households took place. According to Raosoft 2004, a sample size of 560 respondents was envisaged, given a confidence level of 95%, an error margin of 4.08% and a response distribution of 50%. However, the sample size was increased to 610 to ensure that all the bomas were included. The questionnaires were administered proportionately in five main payams, including Lasu111 (18.4%), Yei town 166 (27.5%), Mugwo79 (13.1%), Otogo156 (25.9%) and Tore 91 (15.1%) respectively. A total of 610 respondents across the payams were randomly sampled and interviewed using the structured questionnaires which were proportionately distributed to the various payams then to the bomas and villages. The sampling ensured that all the villages were represented. Households were selected from a random starting point and household members, either male or female, who consented to be interviewed, were selected. Sampling of males and females were alternated to ensure an almost equal representation of both. Both male and female respondents, aged between 18 and 90 years, participated in the study. To complement and contextualize the KAP survey, eight in-depth interviews were held with key informants in charge of HAT related activities from different health facilities, who also happened to have participated in past sleeping sickness programmes. Out of the eight, three of them were former sleeping sickness patients and one had a spouse who had recently been diagnosed with HAT. The purpose of interviewing them was to explore their perspective of community KAP and their individual opinions and experiences (past and present) with HAT. One focus group discussion (FGD) was held with nine male discussants from one of the payams (Otogo). The participants included those who had tested positive with the HAT rapid diagnostic test (RDT), but had not gone for confirmatory testing. This category of discussants was chosen to try and get their perceptions about HAT and the current diagnostic and treatment interventions as well as the challenges they face in completing the referral process for HAT diagnosis. After the interviews, the participants of the FGD were linked with the head of the Yei county health department to ensure they underwent the HAT confirmatory tests. The aim of the FGD was to explore participant’s opinions and experiences with HAT and its past and current interventions. We had envisaged to have more than one FGD but it became a challenge to get the category of people we were keen on having discussions with, coupled with the long distances on rough terrain, and hence we focused more on the key informants who were easier to access.

Before initiation of the study, the study team had a meeting with the Yei county administration team to introduce the study and plan how to conduct the baseline surveys, including mobilizing and sensitizing the community about the study. Research supervisors and assistants were recruited from the community and taken through a one-day training on the basics of conducting the research, including sampling and how to administer the questionnaires. Role plays were carried out to help the research assistants familiarize with the questions in the tool. The questions on attitude using the likert scale gave some research assistants challenges but we managed to put a lot of emphasis in the training on these questions, as well as reviewed all the filled-in questionnaires to clarify any anomalies. Structured questionnaires, composed mainly of closed ended questions focusing on the tsetse and sleeping sickness situation in the study area, and on participants’ awareness, attitudes and practices about symptoms of sleeping sickness, its transmission, diagnosis and management, were administered to all participants. The questionnaires were in English but were translated to the respondents verbally during the interviews. Respondents were asked questions from the questionnaire without having the choices read to them and the research assistants recorded the answers given based on the listed choices. The questionnaire was structured to allow for ease of administration and analysis. However, it had an option of recording anything that did not fall in the given choices, under the category “other” including also a few open ended questions. The questionnaire was based on four main themes, including (1) level of knowledge and awareness about HAT, (2) attitudes and perceptions about HAT, (3) health seeking behavior in relation to HAT, and (4) sources of information about HAT. The theme on health seeking behavior was a hypothetical question given that not all respondents would have experienced HAT. However their responses gave indications of community health behaviour. Given that communication plays a vital role in health campaigns to create awareness, increase knowledge and skills about health conditions and initiate the uptake of health services, it was therefore necessary to also establish the preferred sources and effective channels of communication in the community. The FGDs/key informant interviews were conducted by the first author who is trained in qualitative methodologies while the second author, with training in community public health and work experience in the study area, recorded the notes. The key informant interviews and FGD were conducted in English but a translator helped with translation when necessary. The key themes in the FGD and key informant interviews included: historical perspectives of HAT including myths, common signs and symptoms of the disease, perceptions about HAT, its patients and preferred channels of information dissemination to the community.

Seven out of 610 questionnaires were excluded from the analysis of quantitative data because they were incomplete. Analysis was run on 603 questionnaires which were initially sorted and cleaned and entered in the statistical package for social sciences (SPSS). Descriptive statistics was used to analyse the data using frequencies and percentages. In the analysis, responses related to options that were true of HAT were considered as correct responses, hence indicative of good knowledge of HAT, while responses that were not true of HAT were considered incorrect hence indicative of a gap in knowledge about that specific component. Qualitative data from the FGD and the key informant interviews were analysed using a deductive approach based on already identified themes. Notes from the qualitative data were organized and summarized according to the identified themes and presented in the form of quotes to contextualize the quantitative data.

## Results

### Socio-demographic profile

Out of 603 respondents who participated in the study, 332 (55%) were male and 271 (45%) female, 419 (70%)were between 21 to 50 years old, most (79.6%) had attained a primary level education and below and were mainly crop farmers (75%) (Table 1).

**Table 1:** Socio-demographic characteristics of respondents.

	Frequency (n = 603)	Percentage
**Age**		
18–20	34	5.6
21–30	117	19.4
31–40	178	29.5
41–50	124	20.6
51–60	84	13.9
<60	60	10.9
**Highest level of education attained**		
None	126	20.9
Primary	354	58.7
Secondary	105	17.4
Above secondary	15	2.3
Other	13	0.5
**Main Occupation**		
Crop farming	452	75
Self-employed	64	10.6
Salaried	43	7.1
Casual labourer/ wage employment	21	3.5
Livestock farming	4	0.7
Fishing	3	0.5
None	16	2.7

### Knowledge, attitudes and perceptions about human African trypanosomiasis

Detailed aspects of knowledge about HAT among the communities in Yei are as shown in Table 2. Human African trypanosomiasis in this community, depending on one’s ethnic background, is known as *“ponge”* in Kakwa language and *“ayanlototo”* or *“gilolototo”* in Bari language. These literally translate to *“the disease of sleeping” or “sleeping sickness”*. Most (99%) of the 603 respondents had heard about HAT. Although more than three quarters(85.7%) reported correctly that the disease is caused by tsetse flies, others mentioned incorrect causes such as mosquitoes (3.2%), shaking hands (0.2%), changes in weather (0.2%),and others(1.8%) such as sugar, dirty environment, dirty water and some unknown fly as the causes of HAT. Further analysis in terms of differences in knowledge across the payams, Yei had the most (93%) respondents who gave correct answers, followed closely by Lasu (91%), Otogo (85%), Tore (75%) and Mugwo (72%). The reverse, that Mugwo also had the highest percentage of respondents who gave incorrect answers for causes of HAT, also applies. In terms of gender, more women (43%) than men (25%) gave incorrect responses to causes of HAT while in terms of education, those with at most a primary level of education had higher percentage of people (15%) giving incorrect answers as opposed to those (4%) who had attained at least a secondary level of education.

**Table 2:** Knowledge about HAT among the community in Yei county, South Sudan (n = 603).

Mentioned the following as a sign of HAT**	Frequency	Proportion (%)
Abnormal sleeping*	109	18
Weight gain	90	15
Mental problems*	84	14
Severe headache*	48	8
Increased appetite	31	6
Body weakness*	31	6
Aching muscles*	24	4
Swollen lymph nodes*	24	4
Fatigue*	24	4
Fever*	24	4
Loss of appetite*	24	4
Chancre*	18	3
Others	18	3
**Causes of HAT**		
Tsetse flies*	517	85.7
Mosquitoes	19	3.2
Changes in weather	1	0.2
Eating certain kinds of foods	2	0.3
Shaking hands	1	0.2
Mother to child transmission*	2	0.3
Others	11	1.8
Don’t know	41	6.8
**Pre-disposing activities for HAT**		
Fishing*	254	42.8
Herding*	102	17.2
Farming*	100	16.9
Eating together	35	5.9
Sleeping in the cold	24	5.9
Contact with blood	41	4
Others	9	6.9
**High risk places for HAT**		1.5
Along rivers*	300	50.5
Around bushes/forests*	205	34.5
Grazing areas*	27	4.5
Homesteads	6	1
Everywhere	13	2.2
Others	6	1
Don’t know	37	6.2
**Prevention**		
Clearing bushes around homesteads*	289	48
Putting on light coloured clothes*	97	16
Sleeping under bed nets	91	15
Vaccination	84	14
Applying insect repellants*	42	7

**This was a multi-response question

*Correct response

The respondents were asked about the common signs and symptoms of HAT, and all the symptoms they mentioned were ticked against the choices.

Given the many varied symptoms mentioned a number of times, the percentages under each symptom are low. However, they still illustrate the common signs and symptoms of HAT as known by the community. The general signs and symptoms of HAT, according to the respondents, included abnormal sleep (18%), weight gain (15%) and mental problems (14%). Erectile dysfunction and memory loss were classified in the “others” (3%) category of signs and symptoms. When asked about the three main symptoms of HAT, 21% of the respondents mentioned abnormal sleeping, 20% mentioned severe headache, while 16% mentioned weight gain. However, discussants in the FGDs were in consensus that abnormal sleeping and abnormal itching/rashes were the key signs of HAT. This was also echoed by some key informants.

The main correct activities that respondents mentioned as pre-disposing people to get infected with HAT included fishing (42.8%), herding (17.2%) and farming (16.9%). A few (1.5%) respondents mentioned other incorrect factors, such going to dirty places, sleeping a lot, using dirty water, and walking in the sun. More men (43%) than women (35%) mentioned these incorrect factors. Bathing and washing along the rivers, burning charcoal, walking in the forest, and walking outdoors early in the morning were also mentioned under the ‘other’ category, and are more or less related to correct responses. Correct potential high risk areas for contracting HAT were along rivers (50.5%) and bushes/forests (34.5%). Others (1%) included incorrect information such as cold places, dirty places, near dirty water, and in houses where mosquito nets are not used. Tore payam topped the list with the most respondents (16%) citing incorrect responses in comparison to the other payams, as relates to high risk areas or HAT. Mugwo payam followed by 8%, Otogo (7%), Yei (4%) and Lasu (3%).Key informants consider rivers in the villages as high risk areas for contracting HAT. The main rivers considered as places with a lot of tsetse flies were River Kobo, River Kiju, River Boko and River Tore. For prevention of HAT, respondents mentioned clearing of bushes around the homestead (48%), wearing light coloured clothes (16%), sleeping under bed nets (15%), vaccination (14%), and applying insect repellants (7%).Most (68.3%) respondents reported that men are more prone to sleeping sickness, while 15.8% cited women as most prone. Other respondents (14.4%) did not know which gender is most prone to sleeping sickness.

To establish whether aspects of stigma existed, respondents were asked how people perceive those suffering from HAT and their responses were matched to the choices listed in the questionnaire. Most respondents (71%) reported that HAT patients would get community support and help, 13% reported that they would be avoided, 9% reported that they would be rejected, and 7% mentioned others, including being secluded to eat alone, or being kept in their own rooms. Contrary to the aspect of community support, this quote exemplifies the aspect of possible stigma towards HAT cases. *“When you test positive for sleeping sickness*, *people talk bad of you*, *that you have this sickness so and so…*.*”* (FGD).

One way of gauging the perception of respondents about sleeping sickness included reading several statements to the respondents, and asking them to indicate their agreement or disagreement on a likert scale. Results indicate that a majority (69.5%) of the respondents perceive that HAT affects only adults, close to half (41.8%) of them perceive it to be a contagious (person to person) disease, while others believe that it can be treated by herbalists and witch doctors (11.7%), and that HAT patients can survive without treatment (10%). More women (34%) than men (21%) perceived that HAT has no cure. Other perceptions were that HAT is a killer disease (63%), can be treated in any health facility (36.6%), and that its treatment is expensive (45.5%).

### Health seeking behavior for human African trypanosomiasis

#### Preferred treatment option

Respondents were asked several questions in relation to their/community treatment seeking behavior. The preferred interventions sought by the community for treatment of any general health problem included government health facilities (86.4%), private health facilities run by individuals (7.1%), health facilities run by NGOs or churches (5.5%), traditional healers (1.2%), purchase of medication from shops/chemists, and seeking help from family (1.2%).However, when the respondents were asked where they would seek for help if they suspected symptoms of HAT, a majority (97.2%) would go to a health facility, while 1.3% would visit a pharmacy, 0.3% would go to a traditional healer, and 0.2% would self-treat using herbs (Table 3). Given that currently HAT diagnosis and treatment is offered at designated health facilities, some of the alternative options utilized by the respondents, for example, pharmacies and traditional healers, present an opportunity for them to be considered key stakeholders in the HAT referral system. However, given the limitation of this KAP study, the implication of these alternative forms of treatment options in HAT control strategies were not explored further.

**Table 3:** Health seeking behavior for suspected HAT infection.

Option taken if HAT is suspected		Frequency	Percent
Valid	Go to health facility	585	97.2
	Go to pharmacy	8	1.3
	Go to traditional healer	2	.3
	Pursue other self-treatment options (herbs etc.)	1	.2
	Other (specify)	6	1.0
	Total	602	100.0

Concerning the point at which a patient would go to the health facility if she/he suspected symptoms of HAT, most respondents n = 301 (50.1%) reported that they would go as soon as they realize that their symptoms might be related to HAT. About 23% would go to a facility after symptoms that looked like HAT lasted between 3 to 4 weeks, 14% would go after their own treatment failed, and 11.6% would not go to the health facility. Others (1.7%) had no definite time when they would go to the hospital, and reported that they would either go before the sickness weakens them, or when they have free time. When those who would not go to the health facility after showing symptoms of HAT (n = 70) were asked the reasons for their choice in a follow up question, some of the reasons given included challenges with transport to the facility due to long distance to health facility (46%), cost of services being a barrier (25%), hospital hours overlapping with working hours (5%), negative attitude of health care workers (5%), and lack of trust in medical workers (4%). Others (5%) fear finding out that something is wrong, while 10% were not sure where to go for treatment.

Consensus in an FGD revealed that some people fear the finger prick using a lancet during diagnosis, hence the reason why they would react in various ways as indicated above. Other reasons are illustrated through the following quotes:

*“If you are diagnosed with sleeping sickness*, *you are considered not valuable in the society”* (Key informant).

*“If you are tested positive*, *your male organ will die”* (FGD).

These two quotes relate to the issue of stigma and also to the effects of the disease on an individual, rendering them unable to undertake their daily social and economic roles, hence reduced social and economic value to the society. The aspect of the male organ dying relates to reduced libido, hence inability to be sexually active. Exploring existing myths is important because they can be barriers to effective diagnosis and treatment of HAT. The myth on the male organ “dying” was brought up in a FGD of male discussants who had tested positive with the RDT for HAT but had defaulted to pursue further confirmatory tests. It is important to address such issues using community dialogues.

A historical perspective from the community indicated that in the early days when people did not know much about HAT, many myths were rampant, including being bewitched. Some of the key myths were narrated as follows:

*“If found positive for sleeping sickness*, *you would be taken to Lirangu-Yambio in current South Sudan and would be eaten by the whites*. *In the past they would stay away in hospital from home for six months”*(Key informant).

*“Hospital is poisoning people*. *If taken to Omugo in Uganda*, *you will die”*(FGD).

This aspect relates to the many deaths that would occur in hospital due to people presenting for treatment too late when the disease had taken its toll. A key informant narrated how historically in the early 90s people would be forced, by the chiefs who would be accompanied by the police, to present for screening, and force would be used on those who did not comply. When one was diagnosed with HAT they would be taken to a sleeping sickness treatment centre which was often far away and mainly run by international NGOs, and they would stay there for six months. This included the time for treatment up to the first follow up, because the distances were far and the health personnel needed to confirm that the patients were completely cured before being taken back home. Additionally, due to the long distances, their families were not able to visit them, and given that many would be in the late stage of the disease hence die while undergoing treatment, myths such as the ones stated above were developed to try and explain the unknown, including the use of force to be screened and taken for treatment and the deaths that occurred among the HAT patients while away for treatment.

#### Cost of diagnosis and treatment for HAT

With the knowledge that HAT diagnosis and treatment is free, a hypothetical query on respondent’s perceptions about the cost of HAT diagnosis and treatment was asked. The respondent’s choices were fitted in the most applicable choice ranging from free to very expensive. Most (64%) respondents acknowledged that it is free, while 12.4% mentioned that the costs were reasonable, 5% said it was moderately priced, 15.2% mentioned that it was very expensive,and3.2% did not know the cost.

#### Preferred sources and channels for HAT information

Most (80%) respondents felt well informed about HAT, although majority (95%) of them would like to get more information. The respondents reported that they currently get information on HAT from radio (60%), health workers (27%),newspapers (2.8%), religious leaders (2.8%) and village elders(2.2%). In terms of gender, more (21%) men than women (14%) currently get their information on HAT from village elders, while more women (33%) than men (29%) get their information from the radio. Similarly, more women (33%) than men (31%) reported that they get their information from health workers as well as from family and friends (women 12%, men 6%).

The three most preferred sources of information on HAT by majority of respondents (n = 426) were radio(24%) followed by health workers (15%) and village elders (12%). Other sources that followed were printed materials, including brochures and posters (11%), TV (10%), family, friends and neighbors (8%), religious leaders (6%) and school teachers (4%). Much as billboards and posters did not feature very prominently in the survey, a key informant indicated that big posters with pictures are also preferred, and are better at passing information than written text, instead of t-shirts, which would bring issues if some people got while others did not. However in the word of a key informant, *“talking is best because many people cannot read*. *Some even tear posters”*.

In terms of effectiveness, information from key informants and consensus in a FGD ranked the market as the most effective channel of passing information followed by the church, funeral places and health facilities. Besides these three, women groups, teachers/school children, and headmen/sub-chiefs/administrators of payams were also mentioned as effective channels of communication to the country. In the words of a key informant:

*“The groups mentioned are effective mobilizers, because when they talk, they are trusted and respected. Without this group, you will not be accepted or listened to. After this is successful, then you can use radios. Health education is first passed through other channels, indicating that sleeping sickness is dangerous, then you can use radio, given that everyone has a radio”*.

## Discussion

Given that not all respondents for this survey were necessarily former HAT patients, some of the questions related to practice were hypothetical and hence their responses to these were not based on reality. Additionally, results from KAP surveys are highly descriptive without explaining why people behave the way they do hence needs to be interpreted with caution [13,14]. In this case, ethnographic studies are more applicable to in-depth studies of health seeking behavior, attitudes and practices while placing them in context [14]. KAP surveys have an underlying assumption that there is a direct relationship between knowledge and action. In line with this, the discussions and conclusions should be viewed with this context in mind. However these responses are still valid because they give general indications of what may happen in a real situation of infection with HAT and thus can be used to tailor health education messages. This survey revealed a range of important socio-demographic characteristics of the community in Yei that should be taken into account when developing health education campaigns. Previous studies have shown that socio-economic and demographic factors such as education, age, gender, and main occupation among others, play a critical role in disease epidemiology, and need to be taken into consideration when designing health interventions [21, 22]. Most of the respondents in the present survey had low levels of education or none at all and are also among the group that still had misconceptions about HAT. The low level of education could present a barrier to effective reading and comprehension of written IEC materials. This is similar to findings by [18] in a KAP survey of HAT in the DRC, who concluded that education among other factors was significant in the acquisition of knowledge. Developing IEC materials for such communities requires the use of simple illustrative methods of communication that are easy to conceptualize. Given that more women than men gave incorrect responses in relation to HAT, communication interventions need to take the gender factors into consideration to ensure that public health interventions do not only target either of the genders by virtue of their position in the household or community, but ensures that both men and women are targeted appropriately to ensure inclusivity of all the household members. Some previous interventions on reproductive health have registered low uptake because spouses of the women enrolled in the programs were not included in the study, and hence they stopped their wives from participating [23], while on the other hand, some agricultural projects have left out women, given that they only deal with household heads, who are mostly the owners of the farms[24]. On the other hand, given that the main economic activity in the community in this survey was crop farming, the timing of public health campaigns should take into consideration this socio-economic context to avoid scheduling them during peak seasons when activities such as planting, weeding and harvesting are going on. Findings by [25] showed that engagement in farming activities was one of the barriers to community participation in active screening for HAT, as people were reluctant to interrupt their activities to go for screening.

Findings from this survey show that some misconceptions still exist among a small percentage of people in relation to the cause of HAT based on gender, educational levels and geographic location (payams). These variables also played a significant role as determinants for acquisition of knowledge of HAT in the DRC [18]. Some of the misconceptions about the cause of HAT in the South Sudan study included mosquitoes, shaking hands and sugar. A study by [10] also noted similar routes of transmission of HAT including food, animal blood and water among others in Uganda. On the contrary, in the DRC, Kenya and Uganda, traditional beliefs, sorcery and witchcraft were perceived to cause the disease [26, 27, 28]. Further, Brown (1999) reported that the Azandes, a Central-African people also found in South Sudan, associated the chronic conditions of HAT as those caused by witchcraft. The solution to getting cured was appeasing the spirit of the person who had caused the disease. However how these beliefs/practices could influence conceptualization of HAT may not have been adequately captured in this KAP survey, potentially because of limitations in the study design. However, from inference, if a community’s causal explanation for disease differs from those of a biomedical perspective, there is a greater chance of areas of conflict in preventive practices[29].

Tore and Mugwo payams seemed to have higher percentage of respondents who did not know key information about HAT as opposed to those respondents from Yei municipality and Lasu payams which had more people knowledgeable about HAT. Similar findings were observed by [10] where acceptance of tsetse control interventions varied in relation to duration of experience with previous tsetse control programs. These could relate to previous experience and exposure with HAT and HAT related activities. Field reports from Malteser International BMZ indicate that over the years (2002–2007) the number of HAT cases had been higher in Yei followed by Otogo, Lasu, Tore and lastly Mugwo payams. Presence of HAT cases could signify more HAT related activities in that particular area hence more people getting exposed to HAT related information. Differential geographic knowledge might also emerge from learning about the disease from experiences of correctly diagnosed patients. This carries important implications in designing an effective communication strategy for HAT. Many times in the past, interventions have focused more where cases have been reported. However, findings from this survey show the need to also focus on areas with few cases especially in the face of intensifying surveillance and control of HAT in a sustainable manner, because the few undiagnosed cases who may be infected and not treated act as reservoirs of the disease, hence maintaining the transmission cycle [6]

The community in the present survey had good knowledge about signs of HAT, with sleep reported as the key sign. Abnormal sleep, the characteristic symptom for HAT, has also been noted in several studies [17, 26, 28, 30]. However, using sleep as a key sign of HAT can have a negative effect on passive screening and early detection of the disease, given that the sleep signs manifest in the second or late stage of the disease, and hence one may not present to a health facility for HAT screening until this sign is manifested. Furthermore, as much as the community members indicated that they would seek treatment as soon as they have symptoms, a problem arises in the fact that the few symptoms experienced at the onset of HAT can easily be confounded with other common diseases for which people might not seek treatment for. This contributes to delayed diagnosis and presents a much bigger problem, not only for the individual, but also for the community as the infected person becomes a source of infection for others. Acknowledging that irregular sleep patterns is a classic symptom of HAT that is commonly used in IEC materials and algorithm promoted by national programmes, it is equally important to note that relying on the ‘sleep’ symptom, which was also noted by the respondents as one of the key symptoms of HAT, in the era of elimination, may slow down the progress towards achievement of this goal. Furthermore, relying on this classical symptom may make those who are asymptomatic to remain undiagnosed in the community. This calls for including and stressing the other non-classical symptoms of HAT in community education and sensitization as well as undertaking screening (both active and passive) on a regular basis, given that active surveillance and case treatment have been found to be extremely effective in reducing disease transmission [31].

Fishing, herding and farming as mentioned in this survey to be the key activities that mainly expose people to HAT are in tandem with other reports on the same [32, 33].Other pre-disposing activities mentioned by the respondents related to activities carried out in conducive tsetse habitats. Despite the fact that most respondents knew where they were highly likely to contract sleeping sickness, with places along rivers being the high risk areas, about 10% still held on to various misconceptions such as eating together, using dirty water and walking in the sun. Education and communication interventions need to address such inaccurate information about HAT.

Almost a quarter of the respondents had misconceptions about prevention of HAT, such as sleeping under bed nets and vaccination. This presents a gap of knowledge on how to prevent HAT, given that there is currently no vaccine for the disease, while tsetse flies mostly bite people outside of their houses when they are carrying out activities in tsetse habitats. Myths and misconceptions still exist as shown in the results, however this study only relied on one FGD, hence a limitation that needs to be noted while situating the study in context. Myths such as one’s male/reproductive organs ‘dying’ because they have been diagnosed with HAT, or that if they go to health facilities they will die, still exist among some of the community members as was also noted in the DRC[16]. The historical perspectives presented in this study relate to the 1990s when MSF–France responded to an outbreak of HAT and set up a treatment centre in Omugo. Patients from South Sudan were normally transported to Omugo hospital for treatment and due to the long distances back home, they were kept for six months until they completed their first follow up. Similar observations were also noted in a study on perceptions of sleeping sickness in Uganda [34]. They further also note the pressure that was put on those who refused to go for testing as well as the perception that the whites were coming to eat the community members or poison them in hospital. The aspect of poison could relate to the toxic drug melarsoprol that was previously used to treat the late stage of the disease [33]. Such perceptions still exist and may hinder people from going for diagnosis of sleeping sickness. Collective memory influences community response to disease control programs [34]. There is therefore a need to address such myths and misconceptions, given that misconceptions act as barriers to the adoption of control strategies. They limit people’s ability to change their behavior, and can spread and negatively influence the rest of the community [35]. Just as [34] indicates, it is important to consider whether memories of the past could interfere with current interventions. Given that some of the myths were brought up by community members who had defaulted in completing the diagnostic process after testing positive with RDTs, it is important to take such perceptions into account when working on changing community health/treatment seeking behavior, given that new interventions may trigger some historical myths. Information on the new diagnostic and treatment regimes are therefore key in shaping and dealing with existing myths and perceptions on HAT. As [34] noted, knowledge on the new diagnostic procedures and new treatment approaches which have shorter durations of hospitalization, limited cases of relapses and few side-effects and death, can improve on community response to testing.

The perception that sleeping sickness only affects adults as held by 31% of the respondents is contrary to findings in Tanzania and Sudan, which showed that HAT affects children and adults, as well [1, 18, 36]. Although sleeping sickness occurs more in adults due to their higher exposure to tsetse fly bites, the disease can equally affect children, as long as they are exposed to tsetse fly bites, hence the importance of having IEC materials that show that everyone, the young, the old, both men and women are equally at risk of HAT infections by nature of their activities. This implies also mobilizing all members of the community and not just adults for the screening activities, whether active or passive. The education and communication strategies need to also target the health workers to prompt them in considering children in the differential diagnosis of HAT.

Despite the finding in this survey that a majority (89%) of the respondents perceive HAT as serious, there is still need to communicate the seriousness of the disease to the community so that the few who perceive it as less serious get to appreciate it as a disease of public health importance that leads to death if not treated. When a disease is perceived as less serious, then minimal or no measures at all are taken against it, which can contribute to more infections and subsequent deaths related to the same. As such, clear communication about the seriousness of HAT should be made to enable the community put in place appropriate preventive measures and timely response in case of suspicion of HAT infection. Most of the respondents were sure that HAT can be cured, but a small percentage (6%) did not know or were not sure whether it can be cured. This has implications in health seeking behavior, given that this category of people in the community may not bother to seek medical intervention when HAT is suspected. A majority of people surveyed said they would offer social support to patients with the disease. However, from both the quantitative and qualitative study, aspects of stigma were still present although not widespread. This is an issue that needs to be dealt with when a communication strategy is developed. However, as [37] notes, much as stigma is a powerful element in determining health behavior and a key factor in social exclusion, it is not the only cause of social exclusion and should not be the driver of sensitization campaigns if it is not relevant. Fear, feeling stigmatized, embarrassment, shame and sadness, or hopelessness when one is diagnosed with HAT, are negative reactions that could hinder community members from seeking health services. As such, patients and the community should be encouraged to have courage to go to the hospital in case they realize that they may have contracted HAT, given that drugs for treatment are available and the prognosis is much better if detected early, besides the new/improved testing and treatment approaches in place [2].

Confirmatory diagnosis of HAT can be undertaken in selected peripheral health facilities, while treatment is only undertaken in specific health facilities. Findings from this survey indicate that when people do not know what is causing their illness or HAT related symptoms, they would seek health services from other sources such as chemists, traditional healers, family members and even prayers. This is similar to findings from Kenya and Uganda, which showed that HAT patients utilized other options besides health facilities in the process of seeking health care, leading to delays in accurate diagnosis, and thus presentation in the late stage of the disease[26]. It is therefore important to take into consideration the few people who, in case of a HAT infection, are most likely not to get a correct diagnosis, since the diagnosis and treatment of sleeping sickness cannot be fully accomplished outside the designated health facility settings. Sometimes seeking of alternative sources for treatment may be due to barriers that hinder access to quality health services. Due to the scope and limitations of this study design, in-depth focus on the alternative/ethnomedical perspectives of the community was not adequately captured. However, in the face of integrated strategies to eliminate HAT, it is important to incorporate the alternative sources where people seek treatment, as part of the HAT control strategies by sensitizing them on HAT and making them key stakeholders in community–based referral programmes for HAT control. Costs of services were identified as key barriers to seeking treatment in health facilities. Similar findings have been reported by [25] where payment for HAT in the DRC was perceived as unpredictable, excessive and unfair. While HAT is diagnosed and treated free of charge in public hospitals, only 64% of the respondents were aware of the same. Such information should be widely communicated to the public to create awareness on the same. Some respondents gave monetary value to the cost of treating HAT. However, treatment of HAT is carried out for free, courtesy of donation of drugs by manufacturers, and supply to endemic countries by WHO at no cost [1]. Hence the cost mentioned by the respondents could have been indirect costs, or just perceptions of cost. With the current effort to eliminate HAT, there is need to make people aware that diagnosis and treatment for HAT is free, so as not to hinder the community from seeking diagnosis and treatment when they suspect HAT.

Distance was reported as a barrier to seeking services from health facilities if HAT is suspected. In South Sudan, access to designated HAT screening health facilities was reported to be difficult, given that most villages were located more than an hour’s walk from the health facilities, in addition to poor and unreliable road transport [7, 30]. In the face of intensifying surveillance and control of HAT using the new screening and diagnostic tools and strategies as part of the integrated delivery of primary healthcare, distance to the designated health facilities for HAT need to be taken into consideration. Discordance of the health facilities’ hours of operation with the working hours of some respondents also presented a barrier to utilization of such health facilities by patients. This finding conforms to findings in the DRC[26]. Lack of trust and negative attitude of health workers was reported as a barrier to utilization of health facilities by suspected HAT patients. Issues of trust between health workers and patients have also been raised as barriers to seeking health care or participating in HAT campaigns in the DRC and Uganda [26,38].Health workers therefore need to be trained on how to handle such patients in a friendly manner, in order to create trust between them and the patients.

The preferred sources of information were radio, health workers and village elders, while the most effective places of information dissemination were market places, churches, funeral places, and at health facilities. Similar finding were observed by [38] especially as related to the church and health facilities as key places where information on HAT interventions were conveyed. However, they noted varying responses to this information by ethnic groups hence the importance of taking ethnic variations into consideration. This survey also noted some gender variations in preferred sources of information which are critical to consider in enabling effective dissemination of information. The radio has been hailed as a preferred channel of dissemination of information due to its wide coverage in most areas, rural communities included[39, 40]. It is therefore important that communication strategy for South Sudan take into consideration the preferred sources and identified channels of communication dissemination. Failure to use trusted channels and sources of information may hinder communities from adopting the messages disseminated [41].Village elders have also been recognized as important sources of information, given that they are the gatekeepers of the community and sometimes custodians of community culture, hence the need to be made key stakeholders in community-based disease control programs [34].

This survey has highlighted the need to develop a communication package that is capable of reaching the community, for purposes of awareness creation, and for informing the health seeking behavior of respondents. Providing health care providers and community members in HAT endemic areas with better information about treatment-related side effects would also be beneficial for increasing the uptake of HAT control efforts [15].However one needs to also take into consideration that change in knowledge does not necessarily lead to change in behavior, given that many other factors are equally important in influencing health/treatment seeking behavior [12]. Gender, education level, geographic location, previous experience and exposure to HAT among others, are key aspects to consider and incorporate in developing an effective communication strategy, besides incorporating the gaps in knowledge and practices highlighted in this survey. In addition, health education campaigns should include information that explains the development of HAT, the new/improved testing and treatment approaches and how a late treatment can affect the whole community.

The findings of this survey have highlighted useful information in developing an effective communication strategy and community awareness program that can run hand in hand with the passive and active screening interventions by helping to mobilize and sensitize communities on HAT as part of the bigger goal of elimination of the disease.

## Conclusions

The burden of disease is often a function of factors in the social realm, and this presents the need to examine the social dimensions of health and disease when planning for particular disease-focused interventions. The findings of this survey have important implications in: informing public health approaches to HAT; understanding knowledge gaps in identification of causes of the disease, symptoms, management, treatment seeking; attitudes and practices towards diagnosis and treatment; preferred channels and sources of information.

While there is high level of knowledge about HAT by the community in Yei county, some misconceptions revealed in this survey need to be dispelled, in relation to signs and symptoms, causes, people most susceptible, where one can get infected, where to seek appropriate diagnosis and treatment, and cost of HAT treatment in the hospitals, among others.

A majority of people surveyed said they would seek treatment for HAT only at a health centre. Exploratory qualitative work with key informants and people who tested sero-positive with HAT RDTs, however, suggested that myths still circulating in the popular imagination could influence what people do in practice. Given that community members also have varied health seeking behaviours, including seeking for health services from traditional healers, chemists, shops, and a majority from health facilities, there is therefore need to work closely with shops, chemists and traditional healers to help refer HAT suspects to designated health facilities. This is especially important in the face of conflict situations which may render some designated health facilities inaccessible.

On another front, a majority of people surveyed said they would offer social support to patients with the disease but based on the qualitative data, we should be concerned that some patients may nevertheless experience stigma. These apparently contradictory findings might be explained by limitations of the study design. Using mixed methods that take into consideration a balance of the two methods, qualitative and quantitative will help yield more data that can be used to overcome the limitations of the current study. There is therefore need to develop a communication strategy geared at demystifying the myths and misconceptions, and increasing awareness of accurate and current information on sleeping sickness. This will also help increase the uptake of health services from the health facilities in the different villages.

Based on the findings of this survey, the barriers to seeking healthcare for HAT include distance to the hospital or transport challenges, cost of health care services, myths and misconceptions, patient trust, stigma, incompatibility of hospital operating hours, and daily chores of the community, and poverty. There is therefore need to ensure that even the peripheral health facilities are empowered to handle HAT cases. The radio emerged as a key channel for creating awareness, followed by health workers and village elders. There is therefore need to use them in creating community awareness. The strategies to increase knowledge and awareness as well as uptake of HAT health services requires multi-faceted communication approaches, which should be media-based, community-based and facility-based. There is also need to develop a standard information package on general information about HAT, including information on the new/improved HAT treatment approaches, importance of testing and treating among others. This can be distributed to key community mobilisers, including community health workers, chiefs and village elders for use in social mobilization and public health campaigns. Some apparently contradictory findings from quantitative and qualitative methods might be explained by limitations of the study design, but more research is clearly needed to understand how HAT attitudes and knowledge play out in practice. For example, men’s fear of the disease’s effects on fertility could lead some to avoid confirming positive HAT RDT results; in others this fear might even strengthen their willingness to seek treatment and complete referrals. There is need for further ethnographic research on how ethnomedical beliefs/practices could influence conceptualizations of HAT and hence behavior in its control.

### Limitations

A limitation inherent in most KAP studies that equally applies to this survey is that the findings relate to the reported versus observed practice. In relation to attitude questions, respondents may give answers they believe to be generally acceptable. This needs to be taken into consideration while interpreting the results. The authors worked with local research assistants who were trained on the use of the tool among other basic research concepts. Careful planning and pre-testing and training of the research assistants was carried out to minimize on the cultural gap and help place any unclear issues into context. This KAP survey has a limitation in that one is not able to probe further on the depth of the responses given however, it helped to elicit general information about HAT. The study included the use of a few qualitative methods to try and explore some of the issues in-depth however, the sample used was small hence may not represent a broad range of community perceptions on HAT, much as it gives some insights on the same. We also did not manage to conduct more than one FGD due to constraints of access, hence the qualitative information in this survey was from 8 key informants and only one FGD. However, the findings provide useful data that future researchers can build upon for further studies. The findings of this survey are specific to Yei county, hence cannot be generalized to other areas with varying contexts, although the survey provides useful information on knowledge, perceptions and practices in relation to HAT that can guide future HAT control interventions. This survey was carried out in 2015, and since then, the country has undergone some security upheavals. These have caused many members of the community to move to neighbouring countries, mainly Uganda. The findings of this study especially as relates to health/treatment seeking behaviour and the access to health facilities, therefore need to be situated in the current context. However, the information is still useful in improving interventions on control and management of HAT as well as development of a communication strategy which is still applicable to the South Sudanese communities who are still in the country or those who may be refugees in neighbouring countries as well as in populations with similar settings.

## Supporting information

S1 Checklist Strobe checklist. (DOCX)

S1 Quantitative data Excel data file. (XLSX)

S2 Quantitative data Questionnaire. (DOCX)

S1 Qualitative tool Key informant interview guide. (DOC)

S2 Qualitative tool Focus group discussion guide. (DOC)

S1 Ethical clearance Ethical approval letter. (PDF)
