# Supplementary material for: Knowledge, attitudes and practices about human African trypanosomiasis and their implications in designing intervention strategies for Yei county, South Sudan
Source: PLoS Negl Trop Dis. 2018 Oct 1;12(10):e0006826. doi: 10.1371/journal.pntd.0006826 (PMC6181432; doi:10.1371/journal.pntd.0006826)
Supplement: S2 Quantitative data — (DOCX) [file pntd.0006826.s003.docx]

**APPENDIX 1: QUESTIONNAIRE**

**Accelerating control of Human African Trypanosomiasis (HAT) in Uganda using an integrated strategy**

***HAT Communication Strategy: Sleeping Sickness Knowledge Attitude and practices baseline survey***

*Dear client,*

*The Ministry of Health, Republic of South Sudan together with FIND and Malteser conducts this survey with the aim to understand the knowledge, attitude and practices among members of your community. The result of the study will assist the community and government to institute effective measures to prevent and eliminate sleeping sickness by improving sleeping sickness diagnosis and management. We will appreciate if you can spend 15 minutes talking with us. Your participation is voluntary and you may decide not to participate if you wish; however your participation will be highly appreciated. The information you give us will be confidential, which means that your name will not be mentioned anywhere and the information provided by you will be presented only in a summarized form. It is very important that you respond honestly.* Do I have your permission to continue? Yes 🞏 No 🞏

If yes, proceed Thumb Print/signature of respondent------------------------------------------------------

**CODE QUESTION RESPONSE & CODING**

| **County** | **YEI** |  |  |
| --- | --- | --- | --- |
| **00 Payam** | 1. Lasu 2. Yei Town 3. Mugwo 4. Otogo 5. Tore | **03 Household no** |  |
| **01 Boma** |  | **04 Date of interview (DD/MM/YYYY)** |  |
| **02 Village name** |  | **05 Name of enumerator** |  |

**Part A. Demographic profile of respondents**

1. **Gender**

| Male | 🞏 | 1 |
| --- | --- | --- |
| Female | 🞏 | 2 |

1. **How old are you?**

| 18-20 | 🞏 | 1 |
| --- | --- | --- |
| 21-30 | 🞏 | 2 |
| 31-40 | 🞏 | 3 |
| 41-50 | 🞏 | 4 |
| 51-60 | 🞏 | 5 |
| 60 and above | 🞏 | 6 |

1. **What is your marital status?**

| Single | 🞏 | 1 |
| --- | --- | --- |
| Married | 🞏 | 2 |
| Divorced | 🞏 | 3 |
| Widower | 🞏 | 4 |
| Cohabiting | 🞏 | 5 |
| Separated | 🞏 | 6 |

1. **What is the highest level of education you have completed?**

| None | 🞏 | 1 |
| --- | --- | --- |
| Primary | 🞏 | 2 |
| Secondary | 🞏 | 3 |
| Form five and six | 🞏 | 4 |
| Certificate/ diploma | 🞏 | 5 |
| Bachelor degree | 🞏 | 6 |
| Post graduate | 🞏 | 7 |

1. **What is your MAIN occupation(Work done to earn a living)**

| Crop farming | 🞏 | 1 |
| --- | --- | --- |
| Livestock farming | 🞏 | 2 |
| Fishing | 🞏 | 3 |
| Self employed | 🞏 | 4 |
| Wage employed/casual labourer | 🞏 | 5 |
| Salaried employment | 🞏 | 6 |
| None | 🞏 | 7 |
| Other | 🞏 | 8 |

1. **What is your religion?**

| Christian | 🞏 | 1 |
| --- | --- | --- |
| Muslim | 🞏 | 2 |
| Traditional Religion | 🞏 | 3 |
| Other ( ) | 🞏 | 4 |

1. **What means do you use to reach the nearest hospital**

| Walking | 🞏 | **1** |
| --- | --- | --- |
| Bicycle | 🞏 | **2** |
| Motor vehicle/ Motor cycle | 🞏 | **3** |
| Other (specify) | 🞏 | **4** |

1. **How much time does it take you to reach the nearest health clinic or hospital?(Write time taken to reach the facility)**

| 30 min and less | 🞏 | 1 |
| --- | --- | --- |
| 31 to 60 min | 🞏 | 2 |
| 61 to 90 min | 🞏 | 3 |
| 91 to 120 min | 🞏 | 4 |
| 121 to 150 min | 🞏 | 5 |
| 150 to 180 min | 🞏 | 6 |
| 180 to 210 min | 🞏 | 7 |
| 210 to 240 min | 🞏 | 8 |
| 241 and above minutes | 🞏 | 9 |

**PART B. KNOWLEDGE ATTITUDE AND PRACTICES CONCERNING SLEEPING SICKNESS**

1. **Have you ever heard about sleeping sickness?**

| Yes | 🞏 | 1 |
| --- | --- | --- |
| No | 🞏 | 2 |

**( If yes =continue to no. 2 If No skip to part C)**

1. **Where did you first hear about sleeping sickness?**

| Newspaper | 🞏 | 1 |
| --- | --- | --- |
| Billboards | 🞏 | 2 |
| Radio | 🞏 | 3 |
| TV | 🞏 | 4 |
| Brochures, posters and printed materials | 🞏 | 5 |
| Magazines | 🞏 | 6 |
| Village elders |  | 7 |
| Health workers | 🞏 | 8 |
| Family, friends, neighbors and colleagues | 🞏 | 9 |
| Church/ religious leaders | 🞏 | 10 |
| School/ teachers | 🞏 | 11 |
| Internet | 🞏 | 12 |
| Traditional healer | 🞏 | 13 |
| Patient | 🞏 | 14 |
| Was infected | 🞏 | 15 |

1. **What causes sleeping sicknesses?**

| Mosquitoes bites | 🞏 | 1 |
| --- | --- | --- |
| Tsetse fly bites | 🞏 | 2 |
| Witch craft | 🞏 | 3 |
| Changes in weather | 🞏 | 4 |
| Eating some kind of food | 🞏 | 5 |
| Hand shake | 🞏 | 6 |
| Sharing a meal | 🞏 | 7 |
| Physical contact | 🞏 | 8 |
| Violation of taboos | 🞏 | 9 |
| Mother to child infection | 🞏 | 10 |
| Don’t know | 🞏 | 11 |
| Other specify ( ) | 🞏 | 12 |

1. **Where can one get sleeping sickness from?**

| Bushes and forests | 🞏 | 1 |
| --- | --- | --- |
| Grazing areas | 🞏 | 2 |
| Along the rivers | 🞏 | 3 |
| In the homesteads | 🞏 | 4 |
| Any where | 🞏 | 5 |
| Don’t know | 🞏 | 6 |
| Other (specify) | 🞏 | 7 |

1. **Can sleeping sickness be cured?**

| Yes | 🞏 | 1 |
| --- | --- | --- |
| No | 🞏 | 2 |
| Don’t know | 🞏 | 3 |

1. **How can someone with sleeping sickness be cured? (**Check all that are mentioned**).**

| Herbal remedies | 🞏 | 1 |
| --- | --- | --- |
| Home rest without medicine | 🞏 | 2 |
| Praying | 🞏 | 3 |
| Specific drugs given by health centre | 🞏 | 4 |
| Drugs bought from the shop | 🞏 | 5 |
| Don’t know | 🞏 | 6 |
| Other (specify) | 🞏 | 7 |

1. **In your opinion, who can be infected with sleeping sickness?** (Please check all that are mentioned)

| The poor | 🞏 | 1 |
| --- | --- | --- |
| The rich | 🞏 | 2 |
| Crop Farmers | 🞏 | 3 |
| Livestock farmers | 🞏 | 4 |
| Children | 🞏 | 5 |
| Expecting mothers | 🞏 | 6 |
| Fishermen | 🞏 | 7 |
| Other (specify) | 🞏 | 8 |

1. **Which gender is MOST prone to getting sleeping sickness?**

| Men | 🞏 | 1 |
| --- | --- | --- |
| Women | 🞏 | 2 |
| Don’t know | 🞏 | 3 |

1. **What activity MAINLY pre-dispose people to sleeping sickness?**

| Herding | 🞏 | 1 |
| --- | --- | --- |
| Eating together | 🞏 | 2 |
| Sleeping in the cold | 🞏 | 3 |
| Blood contact | 🞏 | 4 |
| Farming | 🞏 | 5 |
| Fishing | 🞏 | 6 |
| Other (specify) | 🞏 | 7 |
| Don’t know | 🞏 | 8 |

1. **What are the signs and symptoms of sleeping sicknesses?(**Tick all signs mentioned **)**

| Severe headache | 🞏 | 1 |
| --- | --- | --- |
| Loss of appetite | 🞏 | 2 |
| Chancre | 🞏 | 3 |
| Fever | 🞏 | 4 |
| Fatigue | 🞏 | 5 |
| Swollen lymph nodes | 🞏 | 6 |
| Aching muscles | 🞏 | 7 |
| Behaviour change (Mental problem) | 🞏 | 8 |
| Abnormal sleeping | 🞏 | 9 |
| Loss of weight | 🞏 | 10 |
| Convulsions | 🞏 | 11 |
| Don’t know | 🞏 | 12 |
| Other (specify) | 🞏 | 13 |

1. **Which are the THREE MAIN symptoms that distinguish Sleeping sickness from other diseases?**

| Severe headache | 🞏 | 1 |
| --- | --- | --- |
| Loss of appetite | 🞏 | 2 |
| Chancre | 🞏 | 3 |
| Fever | 🞏 | 4 |
| Fatigue | 🞏 | 5 |
| Swollen lymph nodes | 🞏 | 6 |
| Aching muscles | 🞏 | 7 |
| Behaviour change (Mental problem) | 🞏 | 8 |
| Abnormal sleep | 🞏 | 9 |
| Convulsions | 🞏 | 10 |
| Persistent malaria | 🞏 | 11 |
| Don’t know | 🞏 | 12 |
| Other (specify) | 🞏 | 13 |

1. **How can sleeping sickness be prevented?**

| Sleeping under a bed net | 🞏 | 1 |
| --- | --- | --- |
| Clearing bushes around the homestead | 🞏 | 2 |
| Putting on light coloured clothes | 🞏 | 3 |
| Applying insect repellant | 🞏 | 4 |
| Vaccination | 🞏 | 5 |
| By praying | 🞏 | 6 |
| Taking herbal remedies | 🞏 | 7 |
| Nothing | 🞏 | 8 |
| Don’t know | 🞏 | 9 |
| Other (Specify) | 🞏 | 10 |

1. **How do people in your community perceive people suffering from sleeping sickness?**

| Most people reject them | 🞏 | 1 |
| --- | --- | --- |
| Most people are friendly, but they generally try to avoid them | 🞏 | 2 |
| The community mostly supports and helps them | 🞏 | 3 |
| Other (Specify) | 🞏 | 4 |

**Read the statements below and mark appropriately on the table provided and indicate the appropriate code:**

|  |  | **Agree** | **Strongly agree** | **Undecided** | **Disagree** | **Strongly disagree** |
| --- | --- | --- | --- | --- | --- | --- |
| **14** | Sleeping sickness only affects adults |  |  |  |  |  |
| **15** | Sleeping sickness is transmitted through tsetse fly bites. |  |  |  |  |  |
| **16** | Sleeping sickness is contagious. |  |  |  |  |  |
| **17** | Health workers can easily diagnose sleeping sickness. |  |  |  |  |  |
| **18** | Community members can easily identify sleeping sickness |  |  |  |  |  |
| **19** | Treatment for Sleeping sickness is available in the hospitals |  |  |  |  |  |
| **20** | Sleeping sickness can be treated by herbalists and witch doctors |  |  |  |  |  |
| **21** | People suffering from sleeping sickness can survive without treatment |  |  |  |  |  |
| **22** | Sleeping sickness is a killer disease in this area |  |  |  |  |  |
| 23 | Sleeping sickness is not a problem in this village |  |  |  |  |  |
| 24 | Treatment for sleeping sickness is very expensive |  |  |  |  |  |
| 25 | Sleeping sickness can be treated in any health facility in this payam |  |  |  |  |  |
| 26 | Cases of sleeping sickness have decreased in this payam |  |  |  |  |  |
| 27 | Sleeping sickness is a very serious disease |  |  |  |  |  |
| 28 | Sleeping sickness I a very serious disease in this payam |  |  |  |  |  |

**PART C. HEALTH SEEKING BEHAVIORS**

1. **Where do you usually go if sick, or to treat a general health problem?** (Check all that are mentioned)

| Private health facility run by individual | 🞏 | 1 |
| --- | --- | --- |
| Government clinic or hospital | 🞏 | 2 |
| See ta traditional healer | 🞏 | 3 |
| Seek help from the family | 🞏 | 4 |
| Visit the chemist/ pharmacist | 🞏 | 5 |
| Buy pain killers from the shop | 🞏 | 6 |
| Clinic run by an NGO or church | 🞏 | 7 |
| Other (specify) | 🞏 | 8 |

1. **Which is the MOST PREFFERED intervention when one contracts SS?**

| Private health facility run by individual | 🞏 | 1 |
| --- | --- | --- |
| Government clinic or hospital | 🞏 | 2 |
| See ta traditional healer | 🞏 | 3 |
| Seek help from the family | 🞏 | 4 |
| Visit the chemist/ pharmacist | 🞏 | 5 |
| Buy pain killers from the shop | 🞏 | 6 |
| Clinic run by an NGO or church | 🞏 | 7 |
| Other (specify) | 🞏 | 8 |

1. **Why do you prefer the option mentioned in 2 above?**

| 1 | 🞏 | Its cheap/ free |
| --- | --- | --- |
| 2 | 🞏 | They offer quality services |
| 3 | 🞏 | It is the nearest to where I stay |
| 4 | 🞏 | Has a variety of services |
| 5 | 🞏 | Favorable service hours |
| 6 | 🞏 | Friendly health providers |
| 7 | 🞏 | Qualified doctors |
| 8 | 🞏 | Well equipped with lab services |
| 9 | 🞏 | Provide variety of services |
| 10 | 🞏 | Other (specify) |

1. **Which health facilities do you go to when feeling unwell? (List the names in order of most preferred**

| 1 | 🞏 | Hospital |
| --- | --- | --- |
| 2 | 🞏 | HC IV |
| 3 | 🞏 | HC III |
| 4 | 🞏 | HC II |
| 5 | 🞏 | Faith based/ NGO hospital |
| 6 | 🞏 | Dispensaries |
| 7 | 🞏 | Other |

1. **Do you think you can get sleeping sickness? (**Ask the respondent to please explain his/her answer).

| 1 | 🞏 Yes (because…) |
| --- | --- |
| 2 | 🞏 No (because…) |

1. **How would you feel if you found out that you have sleeping sickness?**

| Fear | 🞏 | 1 |
| --- | --- | --- |
| Surprise | 🞏 | 2 |
| Shame | 🞏 | 3 |
| Embarrassment | 🞏 | 4 |
| Sadness or hopelessness | 🞏 | 5 |
| Stigma | 🞏 | 6 |
| Other (specify) | 🞏 | 7 |

1. **What would you do if you thought you had symptoms of sleeping sickness?**

| Go to health facility | 🞏 | 1 |
| --- | --- | --- |
| Go to pharmacy | 🞏 | 2 |
| Go to traditional healer | 🞏 | 3 |
| Pursue other self-treatment options (herbs etc.) | 🞏 | 4 |
| Other (specify) | 🞏 | 5 |

1. **If you had symptoms of sleeping sickness, at what point would you go to the health facility?**

| When treatment on my own does not work **> go to Q#10** | 🞏 | 1 |
| --- | --- | --- |
| When symptoms that look like sleeping sickness signs last for 3-4 weeks **> go to Q#10** | 🞏 | 2 |
| As soon as I realize that my symptoms might be related to sleeping sickness **> go to Q#10** | 🞏 | 3 |
| I would not go to the doctor **> go to Q#9** | 🞏 | 4 |
| Other (specify) | 🞏 | 5 |

**9. If you would not go to the health facility, what is the reason? (**Please check all that apply)

| Not sure where to go | 🞏 | 1 |
| --- | --- | --- |
| Cost/ hospital cost is expensive | 🞏 | 2 |
| Difficulties with transportation/distance to health facility | 🞏 | 3 |
| Do not trust medical workers | 🞏 | 4 |
| Do not like attitude of medical workers | 🞏 | 5 |
| Cannot leave work/duties (Overlapping work hours with medical facility working hours) | 🞏 | 6 |
| Do not want to find out that something is really wrong | 🞏 | 7 |
| Hospital far | 🞏 | 8 |
| Other (please explain)… | 🞏 | 9 |

1. **How expensive do you think Sleeping sickness diagnosis and treatment is in this country?** (Please check one).

| It is free of charge | 🞏 | 1 |
| --- | --- | --- |
| It is reasonably priced | 🞏 | 2 |
| It is somewhat/moderately priced | 🞏 | 3 |
| It is very expensive | 🞏 | 4 |
| Other (specify) | 🞏 | 5 |

**Interviewer: If respondent gives monetary amount, note that amount here:___________**

**PART D. CHANNELS OF INFORMATION FLOW ON SLEEPING SICKNESS**

1. Do you feel well informed about sleeping sickness

| Yes | 🞏 | 1 |
| --- | --- | --- |
| No | 🞏 | 2 |

1. Do you wish you could get more information about sleeping sickness

| Yes | 🞏 | 1 |
| --- | --- | --- |
| No | 🞏 | 2 |

1. What are the **THREE MOST** preferred sources of information that you think can **most effectively** reach people like you with information on sleeping sickness? **Number in order of preference**

| Newspaper | 🞏 | 1 |
| --- | --- | --- |
| Billboards | 🞏 | 2 |
| Radio | 🞏 | 3 |
| TV | 🞏 | 4 |
| Brochures, posters, and printed materials- T-shirts, caps etc | 🞏 | 5 |
| Magazines | 🞏 | 6 |
| Village elders | 🞏 | 7 |
| Health workers | 🞏 | 8 |
| Family, friends, neighbors and colleagues | 🞏 | 9 |
| Religious leaders | 🞏 | 10 |
| School/ teachers | 🞏 | 11 |
| Internet | 🞏 | 12 |
| Other (specify) | 🞏 | 13 |

1. Where do you currently get health information from?

| Newspaper | 🞏 | 1 |
| --- | --- | --- |
| Billboards | 🞏 | 2 |
| Radio | 🞏 | 3 |
| TV | 🞏 | 4 |
| Brochures, posters and printed materials | 🞏 | 5 |
| Magazines | 🞏 | 6 |
| Village elders |  | 7 |
| Health workers | 🞏 | 8 |
| Family, friends, neighbors and colleagues | 🞏 | 9 |
| Church/ religious leaders | 🞏 | 10 |
| School/ teachers | 🞏 | 11 |
| Internet | 🞏 | 12 |
| Traditional healer | 🞏 | 13 |
| Other (specify) | 🞏 | 14 |

1. What source of information do you trust most?

| Newspaper | 🞏 | 1 |
| --- | --- | --- |
| Billboards | 🞏 | 2 |
| Radio | 🞏 | 3 |
| TV | 🞏 | 4 |
| Brochures, posters and printed materials | 🞏 | 5 |
| Magazines | 🞏 | 6 |
| Village elders | 🞏 | 7 |
| Health workers | 🞏 | 8 |
| Family, friends, neighbors and colleagues | 🞏 | 9 |
| Church/ religious leaders | 🞏 | 10 |
| School/ teachers | 🞏 | 11 |
| Internet | 🞏 | 12 |
| Traditional healer | 🞏 | 13 |
| Other (specify) | 🞏 | 14 |

1. How often do you listen to the Radio?

| More than once a day | 🞏 | 1 |
| --- | --- | --- |
| Once a day | 🞏 | 2 |
| Weekly | 🞏 | 3 |

1. What kinds of programs do you like to listen to over the radio?

| News | 🞏 | 1 |
| --- | --- | --- |
| Talk shows | 🞏 | 2 |
| Adverts | 🞏 | 3 |

1. What hours of the day do you usually listen?

| 6:01 to 10:00 AM | 🞏 | 1 |
| --- | --- | --- |
| 10:01 to 14:00 PM | 🞏 | 2 |
| 14:01 to 18:00 PM | 🞏 | 3 |
| 18:01 to 22:00 PM | 🞏 | 4 |
| 22:01 to 2:00 AM | 🞏 | 5 |
| 2:01 to 6:00 AM | 🞏 | 6 |

1. What channels do you usually listen to?
2. What do you think should be done to enable people like you have information about sleeping sickness

- ………………………………………………………………………………………….
- ………………………………………………………………………………………….

**THANK YOU**
