# Supplementary material for: Knowledge, attitudes and practices about human African trypanosomiasis and their implications in designing intervention strategies for Yei county, South Sudan
Source: PLoS Negl Trop Dis. 2018 Oct 1;12(10):e0006826. doi: 10.1371/journal.pntd.0006826 (PMC6181432; doi:10.1371/journal.pntd.0006826)
Supplement: S1 Qualitative tool — (DOC) [file pntd.0006826.s004.doc]

# KEY INFORMANT GUIDE

# Demographics

# Age:

# Sex:

# Occupation:

# Position in the community:

# Level of Education:

# Village: Boma: Payam:

**Theme 1: Current and historical Situation of sleeping sickness in the county**

1. Which are the most affected areas currently with sleeping sickness?
2. What would be contributing to the mentioned changes?

**Theme 2: Community knowledge and practice in relation to sleeping sickness**

1. Probe for knowledge in relation to symptoms, management and control
2. Probe for barriers to seeking health care from medical facilities.

**Theme 3: Channels for dissemination of information in the community.**

1. Probe for preferred and most effective channels/sources.
2. Probe for common language of transmission of information in the community through radio or other media/ common radio stations in the area and their coverage.
3. Probe for the reasons for the answers above.

**THANK YOU FOR YOUR TIME**
