# Supplementary material for: Knowledge, attitudes and practices about human African trypanosomiasis and their implications in designing intervention strategies for Yei county, South Sudan
Source: PLoS Negl Trop Dis. 2018 Oct 1;12(10):e0006826. doi: 10.1371/journal.pntd.0006826 (PMC6181432; doi:10.1371/journal.pntd.0006826)
Supplement: S2 Qualitative tool — (DOC) [file pntd.0006826.s005.doc]

**FOCUS GROUP DISCUSSION CHECKLIST**

# Nature of group:

# Location: Village: Boma: Payam:

# Demographics of participants

| No | Name | Age | Education | Marital status | Main occupation | Position in community |
| --- | --- | --- | --- | --- | --- | --- |
| 1 |  |  |  |  |  |  |
| 2 |  |  |  |  |  |  |
| 3 |  |  |  |  |  |  |
| 4 |  |  |  |  |  |  |
| 5 |  |  |  |  |  |  |
| 6 |  |  |  |  |  |  |
| 7 |  |  |  |  |  |  |
| 8 |  |  |  |  |  |  |
| 9 |  |  |  |  |  |  |
| 10 |  |  |  |  |  |  |
| 11 |  |  |  |  |  |  |
| 12 |  |  |  |  |  |  |

**Theme 1: Historical perspective of sleeping sickness in the community**

- Probe for existing myths

**Theme 2: Common signs and symptoms of the disease**

- Probe on the key signs and symptoms of the disease?

**Theme 3: Perceptions about sleeping sickness and its patiens**

- Probe on any stigma towards the sleeping sickness patients.

**Theme 4: Barriers to health seeking behaviour**

- Probe on reasons why those who test positive for RDT do not go for further confirmatory tests

**Theme 5: Channels of information dissemination**

- Probe on existing channels of information dissemination
- Probe on the most preferred and effective channel and the reasons behind it.

**THANK YOU FOR YOUR TIME AND PARTICPATION**
