# Supplementary material for: Knowledge, attitudes and practices about human African trypanosomiasis and their implications in designing intervention strategies for Yei county, South Sudan
Source: PLoS Negl Trop Dis. 2018 Oct 1;12(10):e0006826. doi: 10.1371/journal.pntd.0006826 (PMC6181432; doi:10.1371/journal.pntd.0006826)
Supplement: S1 Ethical clearance — (PDF) [file pntd.0006826.s006.pdf]

[illegible]

Ministry of Health

15th December,2014

To: **Prof. Joseph Ndungu**
Foundation for Innovative New Diagnostics (FIND)
Campus Biotech, Chemin des Mines 9
1202 Geneva, Switzerland
Email. joseph.ndungu@finddx.org

Dear Professor Ndungu,

RESEARCH APPROVAL LETTER

FEASIBILITY OF ACCELERATING CONTROL OF HUMAN AFRICAN \ TRYPANOSOMIASIS (HAT) IN SOUTH SUDAN USING AN INTEGRATED STRATEGY

The Research Ethical Committee, Ministry of Health, Republic of South Sudan acknowledges receipt of the research proposal submitted by Foundation for Innovative New Diagnostics (FIND) to support study on feasibility of accelerating control of Human African Trypanosomiasis (HAT) in South Sudan using an integrated strategy.

The ethical committee noted that:

1. The study proposes to intensify control of HAT by implementing a novel strategy that combines the use of three new diagnostic tests as part of integrated delivery of health care. ***Integration into the primary health care system*** is unique to South Sudan because in other countries where the same strategy is being implemented, including Uganda, Guinea and Chad, HAT is controlled in vertical programmes.
2. The study will also provide data on the feasibility of using only the RDT and LAMP to identify new cases of HAT, which is likely to make roll out of screening to other regions cheaper and faster.
3. The proposed social research will be used to improve coverage of screening among communities that are at risk. Improved population coverage will accelerate control of the disease by treating the human reservoir of the disease.

Based on the foregoing, the Ministry of Health, Republic of South Sudan hereby approves this collaborative project implemented by FIND, the Government of South Sudan, Malteser International and Jhpiego and therefore assigns Dr. John Rumunu, Director General, Preventive Health Services and Dr. Bior Kuer Bior, Director of Clinical Research and Molecular Diagnostics, Public Health Laboratory as Principal Investigator and Co-investigator respectively.

The project implementation will start as soon as a work plan is approved by the Principal Investigator. All data generated from the study and project implementation will not be published without the consent of the Ministry of Health.

Yours Sincerely,

Dr. Richard Lino Loro Lako
Director General,
Policy, Planning, Budget & Research
Ministry of Health,
Republic of South Sudan

MINISTRY OF HEALTH
15 DEC 2014
REPUBLIC OF SOUTH SUDAN

cc: Dr. Riek Gai, Minister of Health, RSS
cc: Dr. Makur Kariom Undersecretary, MOH, RSS
cc: Dr. Lul, Director General, Public Health Laboratory
cc: Dr. Samson Paul Baba, DG Primary Health Care
cc: Dr. John Rumunu, DG Preventive Health Services
